# Toll-like receptor 9 partially regulates lung inflammation induced following exposure to chicken barn air

**DOI:** 10.1186/s12995-016-0121-x

**Published:** 2016-07-01

**Authors:** David Schneberger, Gurpreet Aulakh, Shankaramurthy Channabasappa, Baljit Singh

**Affiliations:** Department of Veterinary Biomedical Sciences, Western College of Veterinary Medicine, University of Saskatchewan, 52 Campus Drive, Saskatoon, SK S7N 5B4 Canada

**Keywords:** Barn dust, TLR9, CpG DNA, Macrophages, Neutrophils, BAL

## Abstract

**Background:**

Exposure to animal barn air is an occupational hazard that causes lung dysfunction in barn workers. Respiratory symptoms experienced by workers are typically associated with endotoxin and TLR4 signalling, but within these environments gram negative bacteria constitute only a portion of the total microbial population. In contrast, unmethylated DNA can be found in all bacteria, some viruses, and mold. We hypothesized that in such environments TLR9, which binds unmethylated DNA, contributes to the overall immune responses in the lung.

**Methods:**

Using a mouse model, wild-type and TLR9^−/−^ mice were exposed to chicken barn air for 1, 5, or 20 days. Blood serum and bronchiolar lavage fluid was tested against a panel of six TLR9-induced cytokines (IL-1β, IL-6, IL-10, IL-12, TNFα, and IFNγ) for changes in expression. Bronchiolar lavage fluid (BAL) was also tested for macrophage as well as monocyte migration.

**Results:**

There were significant decreases in serum TNFα after a single day exposure in TLR9^−/−^ mice. BAL concentrations of TNFα and IFNγ, as well as TNFα in serum in TLR9^−/−^ mice were also reduced after barn exposure for 5 days. After 20 days of exposure IFNγ was significantly reduced in lavage of TLR9^−/−^ mice. Myeloperoxidase (MPO) accumulation in the lung was reduced at 20 days of exposure in TLR9^−/−^ mice, as was total lavage cell counts. However, Masson’s staining revealed no apparent lung histological differences between any of the treatment groups.

**Conclusions:**

Taken together our data show TLR9 plays a partial role in lung inflammation induced following exposure to chicken barn air potentially through binding of unmethylated DNA.

## Background

Workers in high-intensity livestock operations have been recognized to be at risk for a number of chronic respiratory problems including bronchitis, rhinitis, chronic cough and phlegm, occupational asthma, and organic dust toxic syndrome to name a few [1–3]. Workers in such facilities are exposed to a wide variety of agents such as ammonia, hydrogen sulfide, dust particles, and lipopolysaccharide (LPS) [3, 4]. Even single exposure to such facilities has been shown to elevate a number of pro-inflammatory cytokines in nasal lavages and serum, and induce lung inflammation [5–7]. The mechanisms of these complex in vivo pulmonary responses are not fully understood.

Endotoxin has been targeted as a critical component responsible for many of the lung problems seen in exposure to barn air [1, 7, 8]. More recent work however suggests that within chicken barns, endotoxin-producing gram-negative bacteria comprise only a small fraction of the bacterial species, and that these bacteria may be in the minority as far as total numbers of bacteria present [4]. In contrast, all bacteria and some viruses and mold contain unmethylated DNA in their genomes, which induces a variety of cytokines through binding the TLR9 receptor. Many of these cytokines are similar to those induced by endotoxin, which binds TLR4 [9]. Previous work had shown that DNA from barn dust extracts could induce IL-10 and IL-12p40 [10], however this system exposed isolated peripheral blood mononuclear cells to purified DNA in vitro. This may not be an optimal reflection of barn and in vivo conditions, where significant production of cytokines may occur in the lung from a variety of cell types. Further, such a system does not account for effects of numerous other components in the air that may synergize with or counter responses through TLR9. Therefore, before we dissect the cellular effects in in vitro systems, we need in vivo animal model studies to understand the lung responses to chicken barn air.

Recently, we delineated expression of TLR9 in mouse and human lungs using in situ hybridization and light and electron microscope immunocytochemistry [11]. We have also reported TLR9 expression in cattle, horses, dogs and pigs that spend their lives in barns or other animal containment facilities [12, 13]. This series of studies provided the first data to show in situ expression of TLR9 in airway epithelium, alveolar septal cells and alveolar macrophages. We used this TLR9 expression data to hypothesize that TLR9 promotes in vivo lung inflammation induced by single or multiple exposures to chicken barn air. We tested this hypothesis by exposing TLR9^−/−^ and wild-type mice to chicken barn air for 1, 5, or 20 days. The data show that TLR9 has a partial role in lung inflammation induced following exposure to chicken barn air.

## Methods

### Animals

The experimental protocols were approved by the University of Saskatchewan Committee on Animal Care Assurance and all experiments conducted according to guidelines of the Canadian Council on Animal Care. Breeding pairs of TLR9-deficient mice (C57BL/6 background) were a gift from Dr. Heather Davis and obtained from Taconic. Knockout status was confirmed by PCR on mouse lung tissue. Mice were raised at the Western College of Veterinary Medicine Animal Care Unit. C57BL/6 (wild type) mice where obtained from the Animal Resource Centre at the University of Saskatchewan.

### Experimental exposure

Mice were transported in sealed cages with vents and driven to a cage-based chicken barn in the morning and placed on a shelf approximately 1.8 m (6 ft) off the ground. Mice were kept in barn for 8 h and then returned to animal care facilities at the University of Saskatchewan where they were transferred out of their cages for the evening. Each group consisted of 6 animals per group. Each exposure group was split into two to three subgroups for transport to the barn to ensure that variation of barn or transport conditions on a single day would not account for changes seen in an exposure group. Mice were taken to the barn for 1, 5, or 20 days. The 20 day exposure animals were rested for 2 days after each cycle of 5 exposures to mimic a 5 day work week exposure. Parallel control groups of mice that were transported but not exposed to barn air were transported in separate cages along with the exposed mice.

## Tissue, blood, and lavage collection

At the end of exposure time mice were euthanized (100 mg/kg ketamine + 20 mg/kg xylazine, intraperitoneal injection), and blood was collected by cardiac puncture (Sandoz, Boucherville, QC, Canada). Collection was done approximately 2 h after leaving the barn environment. Blood was separated into serum for cytokine analyses by centrifugation at 2000 *g* for 10 min in Vacutainer tubes (BD, Franklin Lakes, NJ). BAL fluid was collected by flushing lungs with 3 ml of cold HEPES buffer and centrifuged at 400 *g* for 10 min and stored at −80 °C for later use, while the cell pellet was resuspended in 100 μl HEPES and counted with a haemocytometer (Hausser Scientific, Horsham, PA). Cells were resuspended to 800 μl, counted and cytospun onto microscope slides at 1000 rpm for 10 min, and dried overnight before staining with a Hemacolor kit (EMD Chemicals, Mississauga, ON, Canada) according to manufacturer’s protocol. The macrophage and neutrophil counts were converted to absolute numbers. Unfortunately, we lost some of samples from mice exposed five times to chicken barn air during processing and thus cell counts for 5 day exposure were not included in the final analysis.

Lung sections were divided in two, and one half snap-frozen in liquid nitrogen and stored at −80 °C. The other lung was fixed in 4 % paraformaldehyde overnight before dehydration and embedding in paraffin. Lung tissue sections were stained using Masson’s trichrome stain as well as for hematoxylin-eosin, and mounted with cover slips.

## Protein extraction

Frozen mouse lung tissue was homogenized in microcentrifuge tubes using a pestle (Bel-art, Pequannock, NJ, USA). Protein was extracted using an AllPrep DNA/RNA/Protein purification kit (Qiagen, Mississauga, ON, CA) as per manufacturer’s instructions. Protein fractions were saved, quantified, and stored at −80 °C.

## Dust and endotoxin measurement

Dust samples were collected from two random days of exposure on days when animals from 1,5, and 20 day animals were present in the facility. A Sensidyne constant air-flow pump (GilAir-3, Clearwater, FL) run at 2 L per minute was used with a glass fiber filter (1.0 mm binder free type AE; SKC Inc., Eighty Four, PA). The filter was weighed prior to use. Sampling was done at the same location as animal housing in the barn facility for 8 h. Dust was resuspended in 1 ml of water and tested by Limulus Amoebocyte Lysate assay (Cambrex Bioscience, Walkersville, MD) for endotoxin. DNA purification was attempted using a QiaAmp DNA Mini Kit (Qiagen, Mississauga, ON, CA) and quantified using NanoDrop 1000 spectrophotometer (Thermo Scientific, Wilmington, DE)

## Myeloperoxidase assay

Briefly, protein samples were placed on a 96-well plate at several concentrations in phosphate citrate buffer (0.2 M Na_2_HPO_4_ – 7H_2_O, 0.1 M citric acid, pH 5.0) in duplicate along with a recombinant standard. TMB substrate was added to all wells and developed for 2 min at room temperature before reaction was stopped with 1 M H_2_SO_4_ and read at a450.

## Immunohistochemistry

Immunohistology was done on 5 μm thick lung tissue sections. Briefly, after de-paraffinization, rehydration, tissue peroxidase quenching (0.5 % hydrogen peroxide in methanol), and antigen unmasking with pepsin (2 mg/mL 0.01 N hydrochloric acid), the tissue sections were blocked for 1 h with 1 % BSA to block nonspecific binding. Sections were treated with F4/80 (1:75 dilution) and incubated overnight at 4 °C. The next day horseradish peroxidase-conjugated goat anti-rabbit antibody (ab6845, Abcam) was added at 1:100 dilution for 1 h at 37 °C. Color was developed using a color developing kit (Vector Laboratories, Ontario, Canada). Slides were counterstained with methyl green (Vector Laboratories) before mounting. A control was similarly run with omission of the primary antibody or the secondary antibody. We counted F4/80 positive cells in the alveolar septa of lungs to quantify number of septal macrophages. The counts were made in three fields in 4 mice randomly selected from each group in the study at 100X magnification.

## Serum and BAL cytokine bio-plex enzyme linked immunosorbent assay

Cytokines IL-1β, IL-6, IL-10, IL-12, IFNγ, and TNFα were measured using bead-conjugated antibodies and recombinant standards with the Bio-Plex multiplex ELISA assay system (Bio-Rad, Mississauga ON, Canada). Assay was carried out as per manufacturer’s instructions for magnetic bead ELISA and read on a Bio-Plex 200 plate reader (Bio-Rad, Mississauga ON, Canada). Lavage fluid and serum were centrifuged prior to use as described earlier.

## Statistical analysis

All values given were given as mean values with error bars representing standard deviation. One-way ANOVA was preformed to determine significance between groups. Two-way ANOVA was done to assess effect of genotype versus exposure day, with p values generated by Bonferroni post-test.

## Results

### Dust and endotoxin measurement

Dust and endotoxin were measured in the barn over the same period of animal exposure. The average amount of respirable dust for an 8 h exposure was 1.738 μg with a standard deviation of 0.067. The levels of endotoxin measured were 3966 EU/mg of dust. These levels are less than half of those found in a comparable study [14]. Dust levels were also low in comparison. Attempts were made to purify DNA from the samples. An attempt to purify DNA from samples yielded only a single success of 1.065 μg/ml, or 0.6129 μg/μg dust, although this was near the limit of detection.

### BAL total cell numbers

**Fig. 1:**
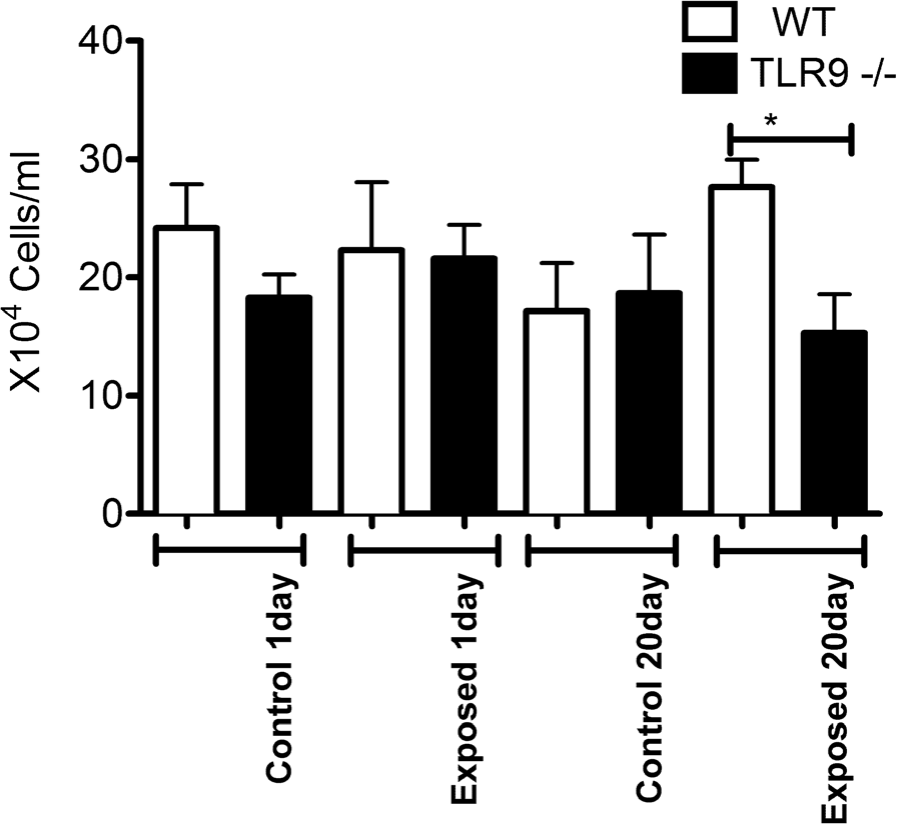
BAL cell counts. BAL from exposed and unexposed WT and TLR9-deficient (TLR9-) mice (*n* = 6) was spun briefly at 400 *g*, supernatant removed, and cells resuspended to 100 μl for counting with hemocytometer. BAL cell numbers were lower in TLR9^−/−^ mice compared to WT mice after 20 exposures (* = *p* ≤ 0.05)

There were no differences in total BAL cell numbers between one day or 20 day control TLR9^−/−^ and WT animals or those exposed for one day to chicken barn air (Fig. 1). The total BAL cell numbers were significantly lower in TLR9^−/−^ mice compared to WT mice after 20 exposures (p ≤ 0.05).

### Neutrophil recruitment into BAL and lung tissues

**Fig. 2:**
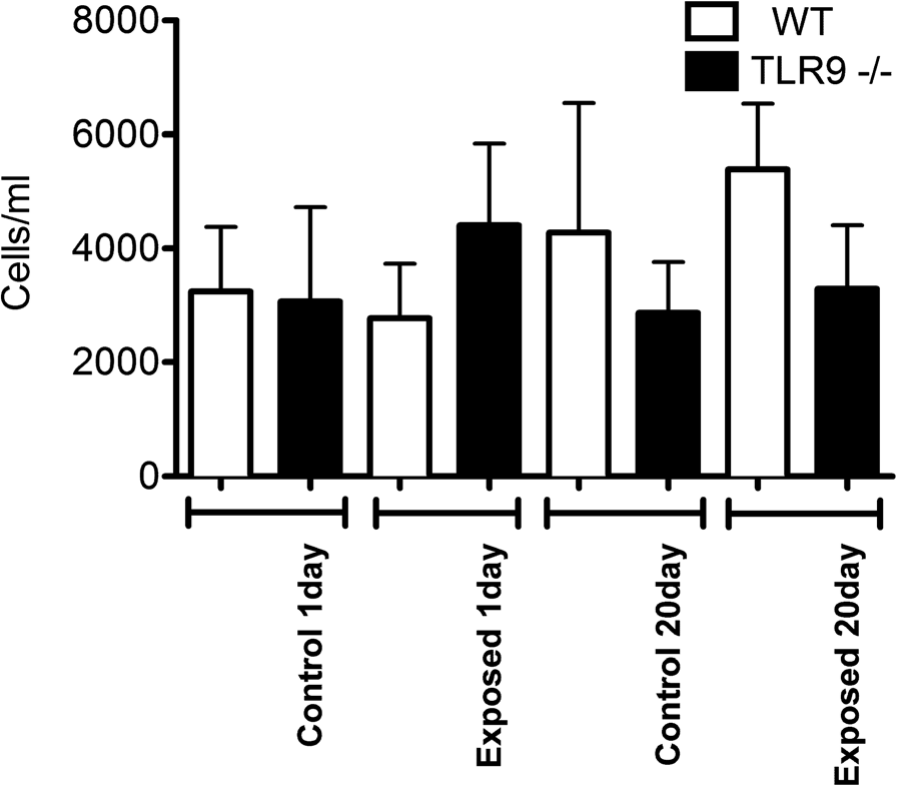
Neutrophil cell counts. BAL from exposed and unexposed WT and TLR9-deficient (TLR9-) mice (*n* = 6) was spun briefly at 400 *g*, supernatant removed, and cells resuspended and stained with a hemacolor kit. Neutrophils were counted and numbers converted to absolute numbers. No significant differences were seen in neutrophil counts between any groups

**Fig. 3:**
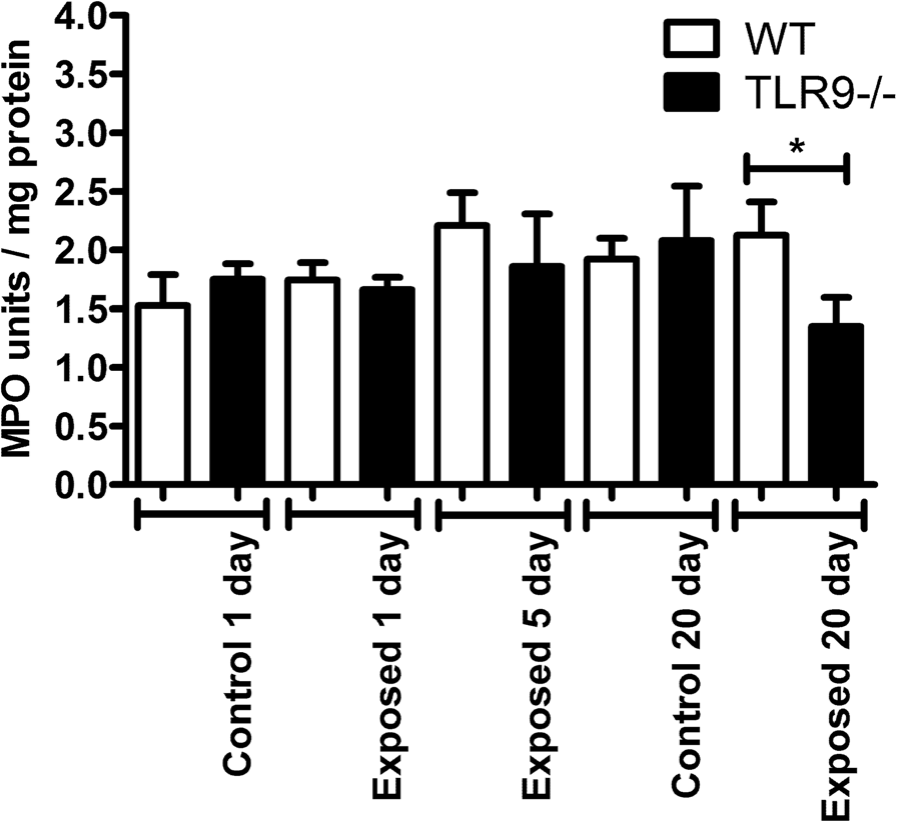
Myeloperoxidase activity assay for lung neutrophil quantitation. TMB substrate was added to protein extracts of whole lung tissue from exposed and unexposed WT and TLR9-deficient (TLR9-) mice and read at a450 after 2 min (*n* = 6). Activity was assessed by comparison to a standard curve. A reduction was seen in 20 day exposed TLR9- mice compared to WT mice exposed for the same time (* = *p* ≤ 0.05)

There were no effects of number of exposures and the strain on the numbers of neutrophils in BAL (Fig. 2). We used an MPO assay as a surrogate for neutrophils trapped in the lung tissues that are not amenable to lavage. MPO assay did not reveal any differences in neutrophil recruitment in lung tissues between the WT and TLR9^−/−^ control mice as well as those exposed once or five times to barn air (Fig. 3). However, after 20 exposures there were reduced MPO concentrations (p ≤ 0.05) in lung homogenates from TLR9^−/−^ animals compared to similarly exposed WT mice. This reduction was not significantly lower than the unexposed control though.

### Macrophage recruitment into BAL and lung tissues

**Fig. 4:**
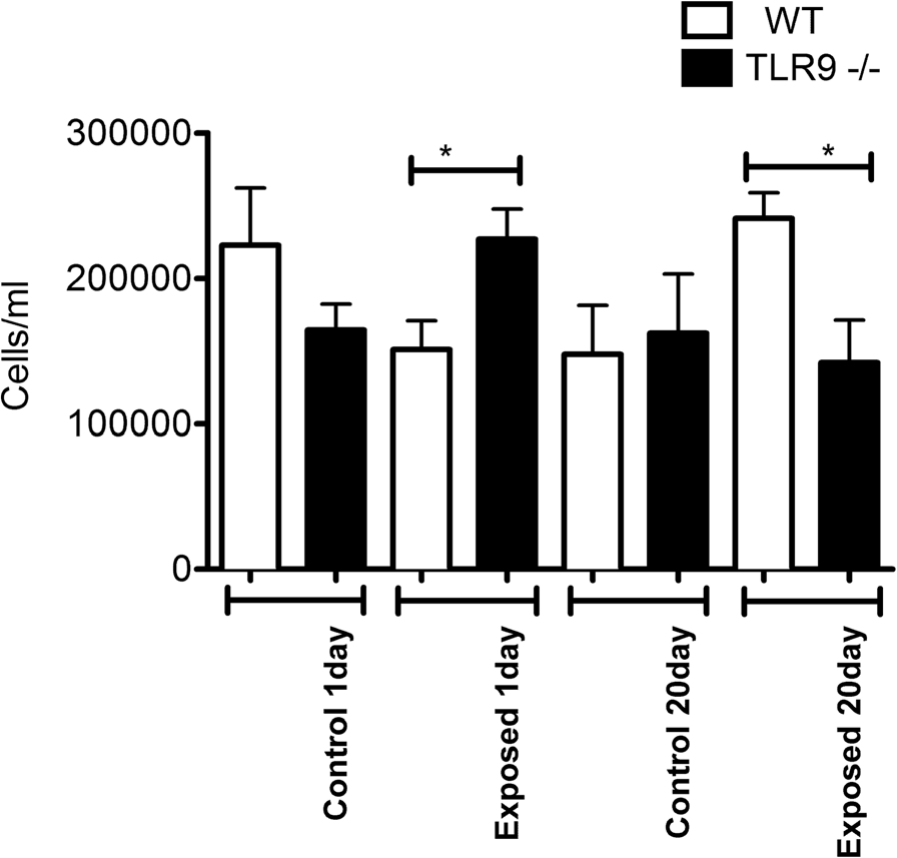
Macrophage cell counts. BAL from exposed and unexposed WT and TLR9-deficient (TLR9-) mice (*n* = 6) was spun briefly at 400 *g*, supernatant removed, and cells resuspended and stained with a hemacolor kit. Macrophages were counted and numbers converted to absolute numbers. Macrophage numbers were higher in 1 day exposed TLR9^−/−^ animals compared to WT mice (* = *p* ≤ 0.05). A similar increase was seen in 20 day control TLR9^−/−^ mice compared to WT mice exposed for the same time (* = *p* ≤ 0.05)

In addition to neutrophils, macrophages are important players in promoting or resolving lung inflammation. There were no differences in macrophage BAL numbers from normal one day WT and TLR9^−/−^ mice (Fig. 4). However, TLR9^−/−^ mice had more macrophages in BAL compared to WT mice after single exposure (p ≤ 0.05). Interestingly, the BAL macrophage numbers were lower in 20 day exposed TLR9^−/−^ mice compared to 20 day wild type exposed animals (p ≤ 0.05).

**Fig. 5:**
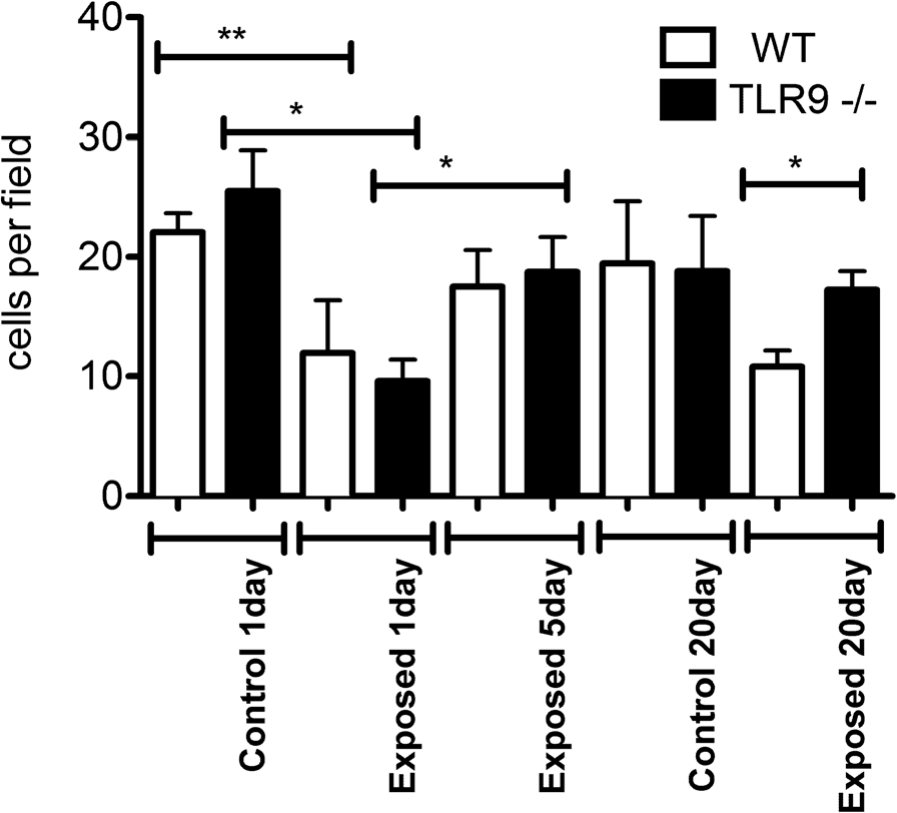
Septal macrophage staining and quantification. Immunohistochemistry with F4/80 antibody was done on lung sections from exposed and unexposed WT and TLR9-deficient (TLR9-) mice. Blinded counts of macrophages were done on five 400X magnification fields on 2 separate lung sections from each animal and averages of these counts used. Septal macrophages were increased in 1 day control TLR9^−/−^ mice compared to WT (** = *p* ≤ 0.01) and TLR9^−/−^ exposed mice (* = *p* ≤ 0.05). After 20 days exposure TLR9- mice had significantly more septal macrophages than exposed WT animals at 20 days (*p* ≤ 0.05)

To determine the number of tissue macrophages in lavaged lungs, we stained lung sections with macrophage F4/80 antibody. Single exposure to barn air induced a significant decrease in the number of septal macrophages in TLR9^−/−^ and wild-type mice compared to unexposed mice at the same time point (Fig. 5, p ≤ 0.05 and p ≤ 0.01). Septal macrophages were significantly increased in TLR9^−/−^ mice after five exposures compared to a single exposure. The number of septal macrophages were significantly higher (p ≤ 0.05) in 20 day exposed TLR9^−/−^ mice compared to similarly exposed WT mice, however these values were still lower than unexposed animals. A two-way ANOVA analysis of genotype versus day of exposure failed to show any significance.

### Histological assessment of lung tissues

**Fig. 6:**
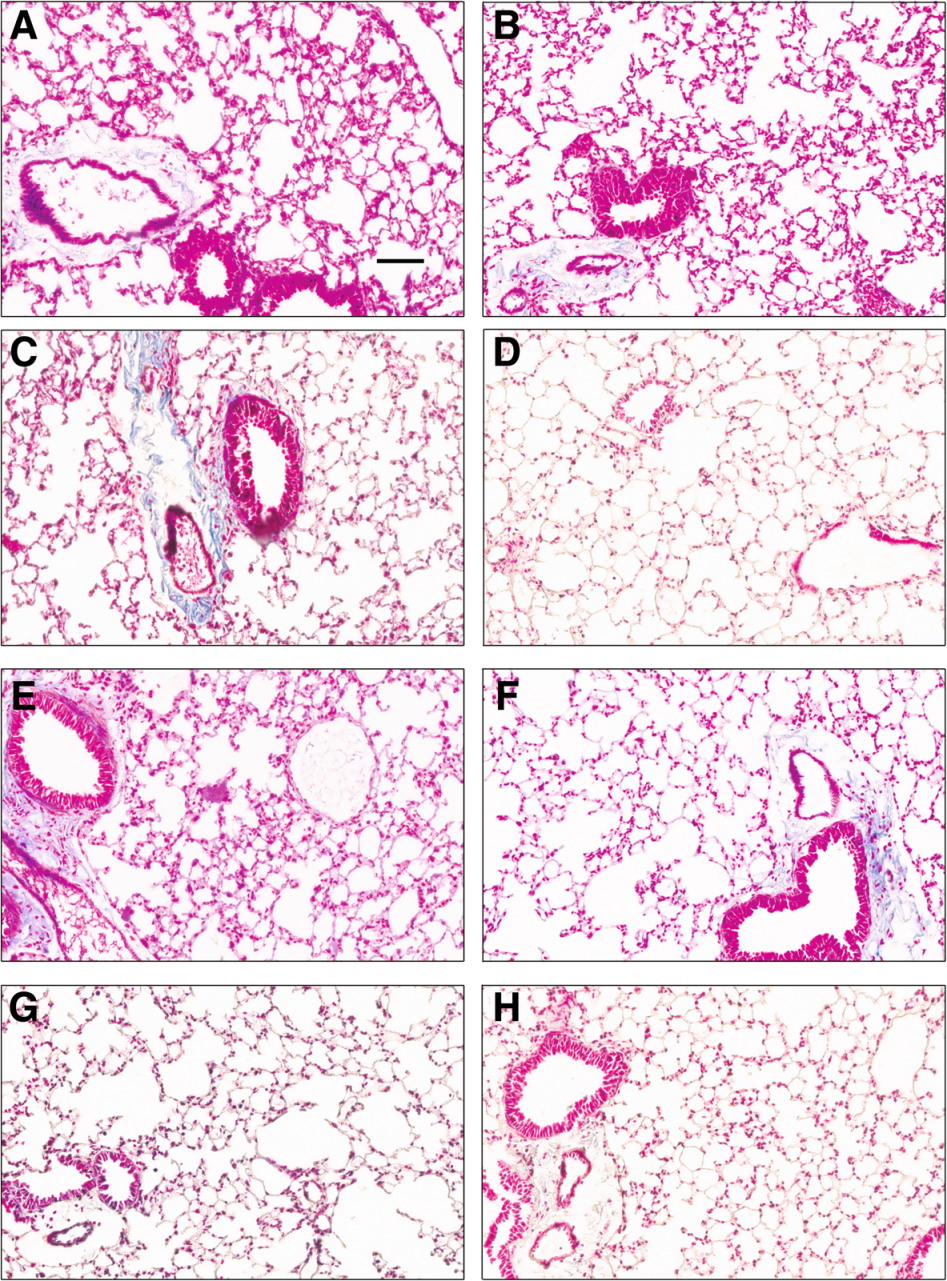
Histopathology of lung samples. Histological assessment shows normal histology in unexposed WT (**a**) and unexposed mutant (**b**). One day exposed WT (**c**) and TLR9^−/−^animals (**d**). WT mice exposed for five days (**e**) showed little difference to the 5 day exposed mutant mice (**f**). Lung sections from both WT (**g**) and mutant mice (**h**) exposed for 20 days showed normal histology. Bar = 100 micron

Lung tissue sections stained with Masson’s stain did not show any apparent differences in extracellular connective tissue (Fig. 6). Histologically, the lung sections from control WT and TLR9^−/−^ mice appeared similar and showed normal architecture. There were no histological differences observed after 5 or 20 exposures to chicken barn air.

### Expression and quantification of cytokines in lung BAL and serum

**Fig. 7:**
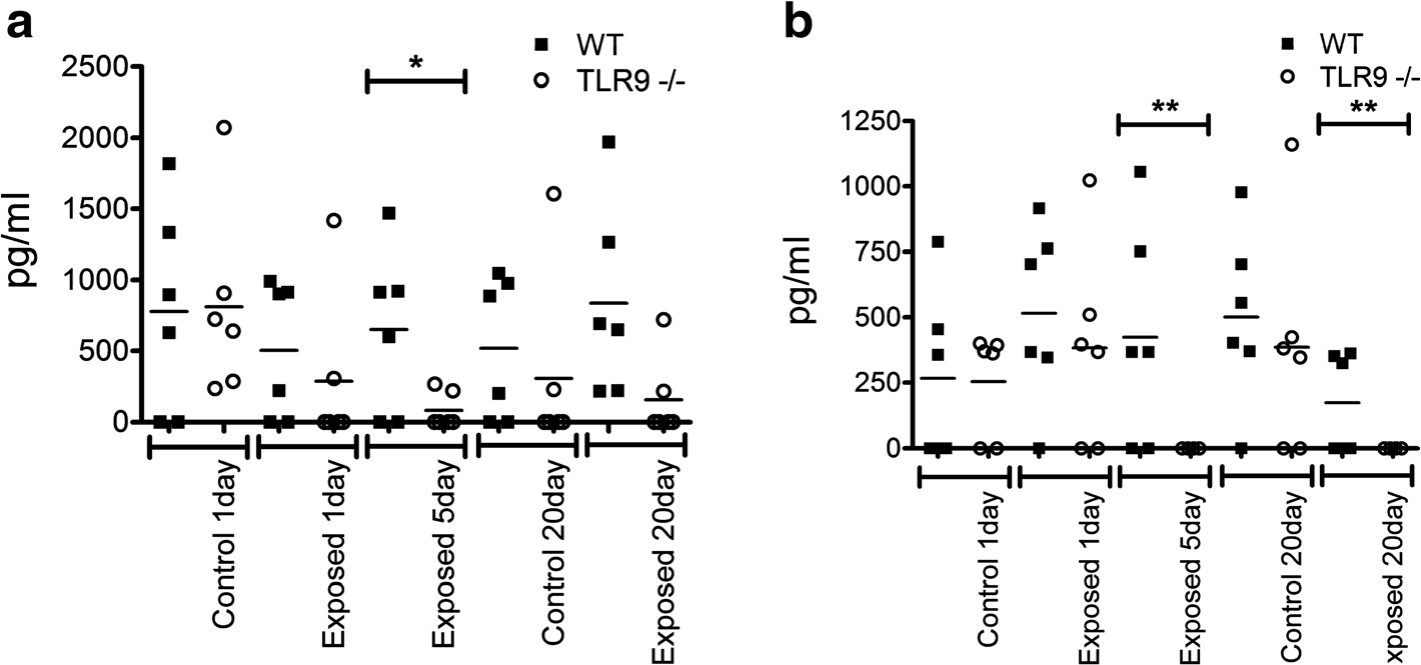
Bio-plex ELISA of mouse bronchoalveolar lavage. BAL was collected by washing lungs 3 times with cold HEPES buffer from exposed and unexposed WT and TLR9^−/−^ mice (*n* = 6). Samples were centrifuged briefly to remove cells and fluid decanted. ELISA was done with antibodies to IL-1β, IL-6, IL-10, IL-12, IFNγ, and TNFα. TNFα (**a**) was significantly reduced after 5 days exposure in TLR9^−/−^ mice compared to WT (* = *p* ≤ 0.06). IFNγ (**b**) was significantly reduced after 5 and 20 days exposure in TLR9^−/−^ mice compared to WT (** = *p* ≤ 0.05)

**Fig. 8:**
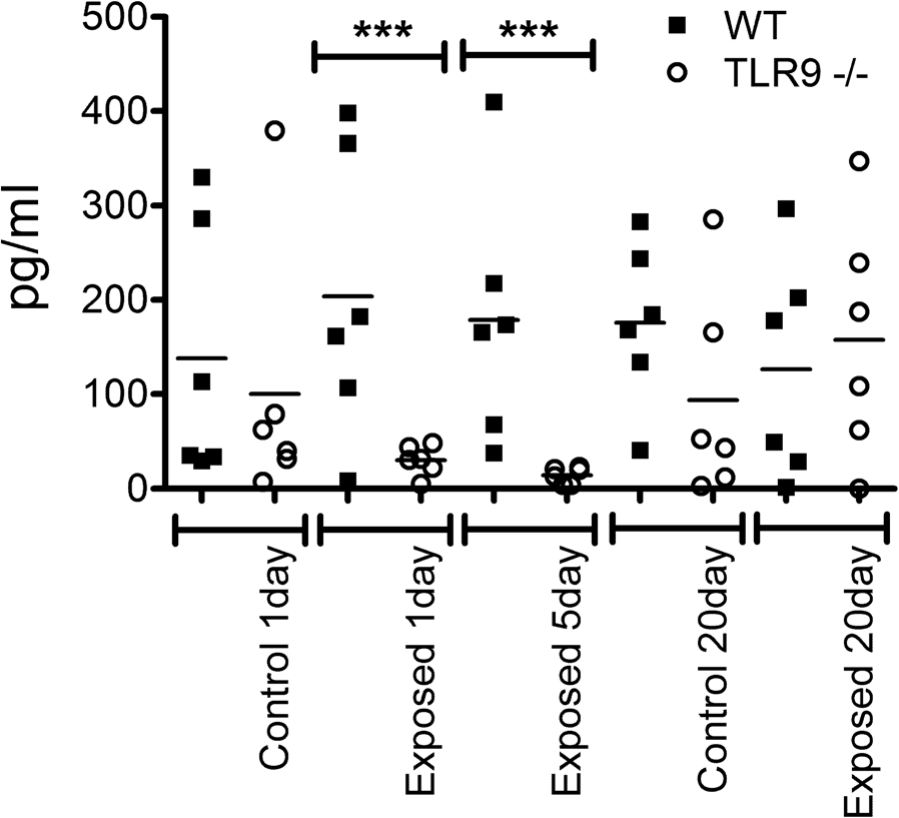
Bio-plex ELISA of mouse serum. Serum was collected by collecting blood via cardiac puncture and then briefly centrifuging to remove cells from exposed and unexposed WT and TLR9^−/−^ mice (*n* = 6). ELISA was done with antibodies to IL-1β, IL-6, IL-10, IL-12, IFNγ, and TNFα. IFNγ was significantly reduced after 1 and 5 days exposure in TLR9^−/−^ mice compared to WT (*** = *p* ≤ 0.01)

A panel of cytokines (IL-1β, IL-6, IL-10, IL-12, IFNγ, and TNFα) were examined in serum and BAL. Of these cytokines significant differences were detected for only IFNγ and TNFα in BAL (Fig. 7) and TNFα in serum (Fig. 8). We do note however that no IL-12 was detected in any samples. There were significantly lower concentrations of TNFα in BAL (Fig. 7a) from TLR9^−/−^ mice compared to WT mice after five exposures barn (p ≤ 0.05). The levels of IFNγ in BAL (Fig. 7b) were lower in TLR9^−/−^ mice compared to WT mice after five and 20 exposures to chicken barn (p ≤ 0.05). There was significantly reduced concentrations of TNFα in serum (Fig. 8) of TLR9^−/−^ mice compared to WT mice after one and five exposures to chicken barn air (p ≤ 0.05).

## Discussion

Chicken barn air has a complex biochemical and microbial composition. Inhalation of chicken barn air is therefore expected to engender a complex inflammatory response in the lungs of humans. Two major components of chicken barn air are endotoxin and unmethylated-CpG DNA. CpG originate from all bacteria, some viruses and molds, and bind to TLR9, initiating an immune response. Previous in vitro work on the effects of purified bacterial DNA on peripheral blood mononuclear cells has alluded to its role in lung immune responses [10]. To better understand the role of CpG in lung dysfunction in chicken barn workers we exposed TLR9^−/−^ mice to chicken barn air for 8 h, to approximately simulate a full work day in a barn facility. The data show a partial role for TLR9, and indirectly CpG, in lung inflammation following exposure to chicken barn air.

The cell numbers in BAL are used as an indicator of lung inflammation [15]. As indicated in Materials and Methods, we unfortunately lost some of the samples from five day exposure group and omitted this group from BAL cell analyses. Our data showed no differences in total BAL cell counts between the one and 20 day control and one day exposed WT and TLR9^−/−^ mice. After 20 exposures however, TLR9^−/−^ mice had significantly lower total BAL cell numbers compared to WT mice. There were no effect of TLR9 on neutrophil numbers in BAL. Also, after 20 exposures, TLR9^−/−^ mice showed higher numbers of septal macrophages compared to WT. Interestingly, the MPO content, indicative of neutrophils left in the lung tissues after BAL has been performed, was also lower in the knockout mice compared the WT after 20 exposures but not after one or five exposures. However, lack of perfusion of the lungs may also be responsible for some of the variation. Taken together, these cellular and histological data suggest lower lung inflammation in TLR9−/− mice compared to WT mice in longer term exposure.

The results of the current study are in contrast to the effect of pig barn air where significant increase in total neutrophil and macrophage numbers in BAL was noticed after a single exposure [16]. The reasons for the differences are not addressed by our experiments but could be related to the higher amount of endotoxin in the air in pig barn because deficiency of TLR4 resulted in significant reduction in total, neutrophil and macrophage BAL numbers compared to the WT animals exposed to pig barn air [5, 7]. There are also likely differences in the total exposure doses found in each barn which could also account for these changes. Second, the differences could be related to the microbial content in chicken and pig barns as even the WT animals didn't show acute inflammation as experienced after exposure to pig barn air. The migration of inflammatory cells into alveolar spaces is regulated through development of a chemotactic gradient produced through production of chemokines such as IL-8, MIP-1 and MCP-1 by airway epithelial cells and alveolar macrophages stimulated by inhaled molecules such as endotoxins [17]. The maintenance in the number of macrophages which resolve and modulate inflammation in the BAL of TLR9^−/−^ mice compared to WT mice following one exposure suggests a modulatory role for TLR9. The early increase in macrophages without any effect on neutrophils or total cells in the BAL in TLR9^−/−^ mice is interesting when combined with fewer tissue neutrophils but comparable tissue macrophages after 20 exposures to chicken barn air. Although neutrophils migrate during the early phase of acute lung inflammation, their migration may continue over a longer period of time in chronic inflammation stimulated through repeated exposures in diseases such as COPD [18, 19]. The data suggest that deficiency of TLR9 dampens lung inflammation induced by exposure to chicken barn air.

The expression of inflammation including recruitment of neutrophils and macrophages is regulated through adhesion proteins and inflammatory mediators [20, 21]. To understand the role of inflammatory mediators, we examined the expression of IL-1β, IL-6, IL-10, IL-12, IFNγ, and TNFα in BAL and serum of mice in our study. These cytokines are produced following ligation of bacterial DNA by TLR9 [22, 23]. We did not find any differences in control or exposed WT or TLR9^−/−^ mice for IL-1β, IL-6, IL-10, and IL-12. This would suggest that while the barn may be an environment known to induce inflammation in the lung, changes in many individual cytokines may not be significantly elevated, or that responses are too variable. While IFNγ was reduced in BAL from TLR9^−/−^ mice after 5 and 20 exposures, TNFα was reduced only after 5 exposures. The levels of IFNγ were lower in the serum of TLR9^−/−^ mice compared to WT mice exposed once or five times to chicken barn air. Although there were differences in IFNγ and TNFα in BAL fluid between WT and TLR9^−/−^ mice after five exposures, there were no differences in the numbers of cells recruited into alveoli after one or five exposures. There were however more septal macrophages in TLR9^−/−^ mice compared to WT mice following five exposures. Because macrophages play a role in resolution of inflammation [21], reduced levels of TNFα along with increased numbers of macrophages may indicate a pro-inflammatory role for TLR9 in lung inflammation induced following exposure to chicken barn air. Because TNFα promotes expression of adhesion molecules [24], reduced levels of TNFα may have also contributed to reduced migration of neutrophils in lungs of TLR9^−/−^ mice compared to WT mice following 20 exposures to chicken barn air. However, there were more septal macrophages present at the same time. TNFα produced by activated airway epithelial cells and alveolar macrophages is an important regulator of lung inflammation as well [24]. Because of the role of TLR9 signaling in induction of pulmonary IFNγ expression [25], the reduced levels of IFNγ in the knockout mice is understandable. However, the effects of reduced expression of IFNγ on the inflammation phenotype in our experiments are not apparent. The role of IFNγ as a modulator of Th2 immune response is something that we need to address in future experiments. It is known that IFNγ is reduced in many cases of asthma [26] and in cases of endotoxin exposure [27]. This is typically associated with a more Th2 cytokine profile. Pig and cattle farm workers had higher incidence of asthma compared to other farm workers [28] which has been attributed to higher endotoxin levels.

The lack of pronounced differences in lung inflammation in WT mice compared to TLR9^−/−^ mice may be due to impaired expression of TLR9 in murine alveolar macrophages [29]. Because alveolar macrophages play critical roles in lung inflammation induced following inhalation of microbes or their products [30–32], impaired TLR9 expression may explain lack of distinct differences in inflammation between WT and TLR9^−/−^ mice. While recognition of endotoxin is still intact in both mouse strains, the differences in lung inflammation are rather subtle. This however does not inform us of the reasons for lack of differences between WT control and one or five day exposed mice. This issue needs to be addressed through comparison of lung inflammation in mice in which alveolar macrophages are depleted prior to their exposure to barn air.

One possible explanation for both BAL and septal macrophage results is that the septal macrophages exert an anti-inflammatory effect on the lung, leading to fewer BAL cells. There is evidence for this in a recent study that showed that signaling through TLR4 and TLR9 in septal macrophages induced expression of cytokines such as IL-10 and generally induced an anti-inflammatory response [33]. Yet another possibility is that migration of macrophages into the alveolar space requires a transition through the alveolar septa [34]. If this is the case then a reduction in macrophages at one time point in the septa being mirrored by an increase in the BAL could be a reflection of the increased movement of these cells into the alveolar space as appears to happen in our experiments. This however still raises the question of the reason for such an increased migration of macrophages.

The response of cellular influx in and out of the lung with TLR9 inhibition is quite intriguing and somewhat perplexing. We suspect that within the first day of exposure lack of TLR9 signaling reduces the rate of out-migration of alveolar macrophage to tissue and lymph nodes [35]. The general reduction in exposed group interstitial macrophages which are known to secrete anti-inflammatory cytokines to the same stimuli [33] suggest migration in and out of BAL may be more likely at this point.

By day 20 however TLR9−/− animals are seeing increases in these interstitial macrophage populations, suggesting a less permissive migration scenario. At this point we see this reflected in reduced total BAL cells, BAL macrophages, and tissue neutrophils. As such, we would hope to do more work on the effects of dusts and TLR9 signaling in interstitial macrophages to better determine their role in lung cellular migration.

Lung responses to bacterial DNA will depend on the amount of inhaled DNA. Attempts to purify DNA from barn dust from a previous experiment typically produced around 1 μg of DNA from a filter kept in a similar barn for 8 h (unpublished observations). Of this a portion of recovered DNA will be methylated eukaryotic DNA, further reducing the amount of stimulatory DNA. Therefore, the predicted exposure to bacterial DNA in the barn is probably quite low, certainly in comparison to what is used in in vitro trials [10]. However, as has been mentioned, dust and endotoxin levels in this facility were lower compared to a wider survey [3]. We would thus predict a greater effect of bacterial DNA in many facilities compared to what is shown here. Although some differences between WT and TLR9^−/−^ mice were noticed after single exposure, the divergence in immune responses between mouse populations at 5 days or later may indicate a requirement for exposure to a sufficient dose of stimulatory DNA that the mice do not see in a single day. Indeed, as Roy and colleagues found, 10 μg of barn dust induced detectable responses [10], which would be approximately the dose encountered by the mice after 5 exposures. This accumulated effect though would have to result from continued exposure, not an accumulation of DNA within the tissue, as other studies have shown that internalized DNA is rapidly degraded [36]. What this suggests is that a low unmethylated DNA dose (perhaps 1 μg/day) may cause changes to lung cytokine secretion, especially in the context of stimulation or challenge by other pro-inflammatory molecules or organisms (in this case endotoxins and/or proteoglycans and particulate dust). Another consideration is what happens the CpG DNA in absence of TLR9 receptor? To what degree could other potential sensing mechanisms be altered by CpG DNA not binding to TLR9?

We do note that a study like this has a number of limitations that must be recognized. First, dust and barn conditions are likely to differ between specific facilities, and moreso between facilities such as swine and chicken facilities, though there is clear evidence of worker lung problems in both as discussed earlier. Second, this study was limited to infiltration of cells and cytokine expression in the lung, possibly ignoring other potential markers of lung inflammation. Even in the cases of exposure there was not a significant increase of inflammatory cells and cytokines above controls animals of the same strain, so there was not a clear indication on inflammation in mice over the course of the experiment. This having been said however, there were clear changes in cytokine expression, macrophage numbers, and MPO concentration in the lungs of mice, with most of these changes being present after a longer term exposure to barn air.

## Conclusions

In conclusion, while we saw little change to BAL cell populations and cytokines in mice that had been exposed to chicken barn air for 1, 5, or 20 days, there were subtle changes in TLR9−/− animals. In TLR9−/− animals we detected changes to total BAL cells, and alveolar macrophage numbers by 1 day exposure, and still at 20 days. Septal macrophages were also increased in the TLR9−/− animals by 20 days exposure, in a pattern opposite to that seen in alveolar macrophages. There were also reductions in TNFα and IFNγ in the TLR9−/− knockout mice. These results suggest that TLR9 plays a role in the innate immune response to chicken barn air exposure that enhances indicators of inflammation (TNFα, IFNγ, MPO) at later time points (5 day or 20 day exposures). However, the full effects of TLR9 may be more complex as shown by increased BAL cell numbers and alveolar macrophages after single day exposure, and increased septal macrophages in these same animals at 20 days.

## Abbreviations

BAL, bronchoalveolar lavage; LPS, lipopolysaccharide; MPO, myeloperoxidase; TLR, toll-like receptor
